# Double trouble - dual outflow tract obstruction in congenital heart disease: a case report

**DOI:** 10.1186/s12872-024-03842-x

**Published:** 2024-04-01

**Authors:** Kanchan Maggo, Aanchal Bhayana, Pranav Gupta, Vidushi Gupta, Animesh Verma, Amita Malik

**Affiliations:** 1 Department of Radiology, VMMC and Safdarjung Hospital, New Delhi, India; 2 Department of Radiology, GB Pant Hospital (GIPMER), New Delhi, India

**Keywords:** Double chambered right ventricle, Subaortic membrane, Outflow tract, Case report

## Abstract

**Background:**

Double chambered right ventricle is a rare congenital heart disease that is characterised by the presence of an anomalous muscle bundle that divides the right ventricle into a low pressure superior (distal) chamber and a high pressure inferior (proximal) chamber. It is found in association with a ventricular septal defect in 90% cases with other associations being tetralogy of Fallot, transposition of great vessels, atrial septal defect and Ebstein’s anomaly. On the other hand, subaortic membrane is a form of discrete subaortic stenosis that is characterised by a membranous diaphragm in the subvalvular location of the left ventricular outflow tract. Both of these entities are responsible for causing subvalvular outflow tract obstruction. The occurrence of double chambered right ventricle in association with subaortic membrane is an extremely rare entity with only a few case reports available in the literature.

**Case report:**

A 13-year-old male child with history of chest pain and palpitations presented to the outpatient department of a tertiary care center. Transthoracic echocardiography revealed a subaortic membrane producing a pressure gradient across the left ventricular outflow tract with dilatation of the right atrium and right ventricle which could not be fully evaluated on echocardiography. Cardiac computed tomography was then performed which additionally revealed an anomalous muscle bundle coursing across the right ventricle from the septum to the subinfundibular region creating a double chambered right ventricle. The patient was then taken up for reconstruction of right ventricular outflow tract and resection of subaortic membrane.

**Conclusion:**

Right and left outflow tract obstructions are rare congenital lesions which when seen in combination, become even more infrequent. Echocardiography is a robust tool that detects turbulent flow to identify such lesions. However, poor acoustic window may sometimes result in missing these lesions and computed tomography in such situations can play an important role in detection as well as complete preoperative imaging evaluation.

**Supplementary Information:**

The online version contains supplementary material available at 10.1186/s12872-024-03842-x.

## Background

Congenital heart diseases have an incidence rate of 17.9 per 1000 population worldwide according to the 2017 Global burden disease study [1]. In the paediatric population, subvalvular causes of outflow tract obstruction are much more common as compared to adult population, where they are found much less frequently. Double chambered right ventricle (DCRV) is a rare congenital heart disease that is responsible for subvalvular right ventricular outflow tract (RVOT) obstruction with an incidence rate of only 0.5-2% [2]. This entity is characterised by the presence of an anomalous muscle bundle in mid portion of right ventricle that divides the chamber into a high pressure inlet portion and a low pressure outlet portion with resultant RVOT obstruction. It is usually associated with other congenital heart diseases such as Tetralogy of Fallot, transposition of great vessels, ventricular septal defect (VSD), atrial septal defect and Ebstein’s anomaly to name a few.

Subaortic stenosis is the most common cause of subvalvular left ventricular outflow tract (LVOT) obstruction with various types of subaortic stenosis outlined in Table 1 [3, 4]. Of these, subaortic membrane (SAM) is the most common entity causing discrete subaortic stenosis. SAM is a rare congenital heart anomaly that is characterised by a membranous diaphragm in the subvalvular region of LVOT. It causes variable degrees of LVOT obstruction depending on the thickness of the membrane and presence of fenestration. SAM is a type of fixed anatomical abnormality and needs to be differentiated from a dynamic functional abnormality such as septal hypertrophy in hypertrophic cardiomyopathy.

**Table 1:** Anatomical types of subaortic stenosis

Type	Description	Location
Type 1	Subaortic membrane	Cranial portion of LVOT
Type 2	Fibromuscular ridge	Few centimetres below aortic valve
Type 3	Irregular fibromuscular additional tissue	Just below aortic valve
Type 4	Fibromuscular tunnel like narrowing of LVOT	Entire LVOT

DCRV is more likely to present with an associated VSD than to occur as an isolated entity. Its association with SAM has rarely been reported with few case reports described so far [5, 6]. In this case report, the authors demonstrate an extremely rare case with concomitant occurrence of DCRV and SAM producing dual chamber outflow tract obstruction. DCRV in this case occurred in the absence of a VSD.

## Case presentation

A thirteen year old boy presented to the cardiology outpatient department of a tertiary care hospital with complaints of chest pain and palpitations for six months. The patient also complained of breathlessness which aggravated upon physical exertion and was relieved by resting. The patient had no history of recurrent lower respiratory tract infections or history of prior hospitalisation. No significant comorbidities were present and no relevant family history was provided. Laboratory investigations were normal. On chest examination, bilateral air entry was normal while on cardiac examination, a low-pitched ejection systolic murmur was appreciated in the second and third left parasternal spaces. Plain frontal chest radiograph of the patient revealed mild cardiomegaly. The patient underwent transthoracic echocardiography which showed a suspicious echogenic membrane below the aortic valve with flow acceleration seen across the LVOT on continuous wave Doppler having a mean gradient of 35 mmHg, peak gradient of 55 mmHg and peak velocity of 3.7 m/s. Additionally, right atrium and right ventricle were found to be dilated and flow acceleration with mean gradient of 40 mm Hg was observed across RVOT as well which was limited by poor acoustic window. For further structural information and complete preoperative evaluation, the patient was advised to undergo cardiac computed tomography (CT) examination.

**Fig. 1:**
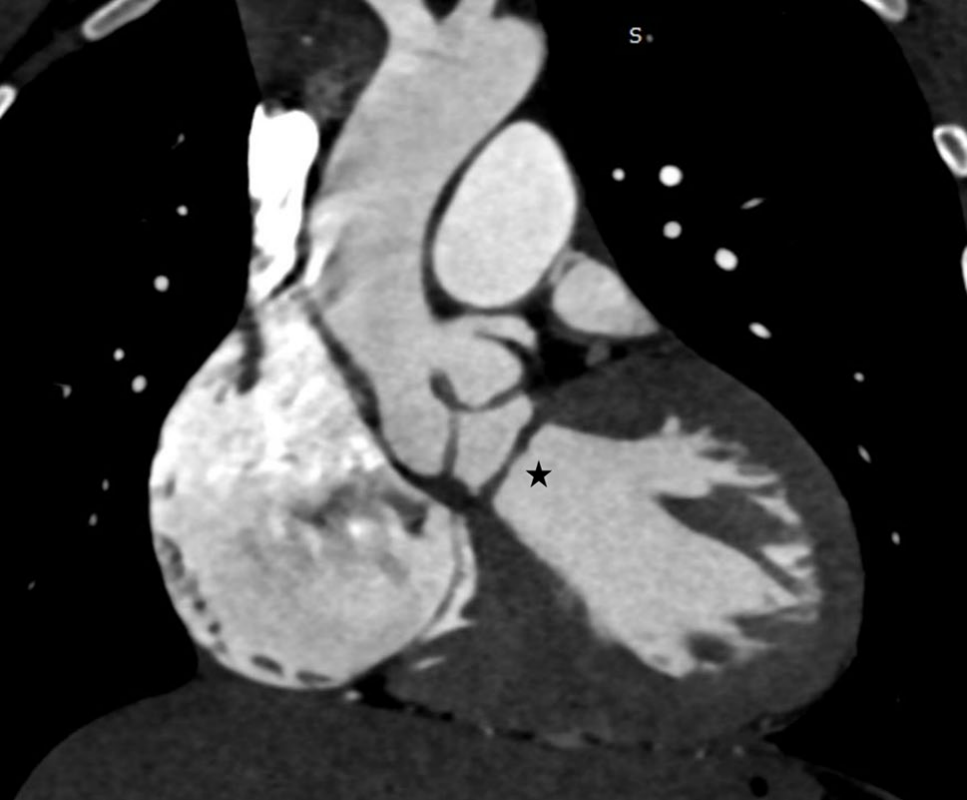
Cardiac CT showing subaortic membrane in the left ventricular outflow tract (star) beneath the aortic annulus

**Fig. 2:**
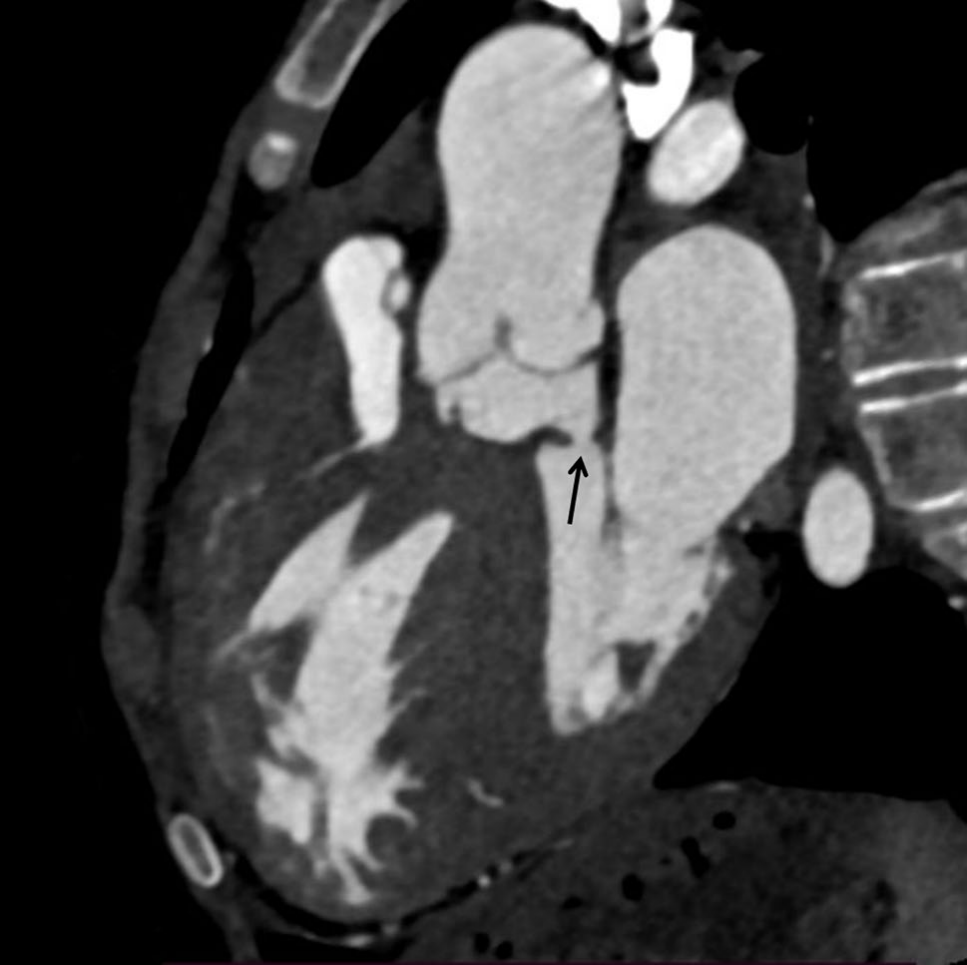
Cardiac CT oblique sagittal section showing subaortic membrane with a small fenestration (thin arrow)

**Fig. 3:**
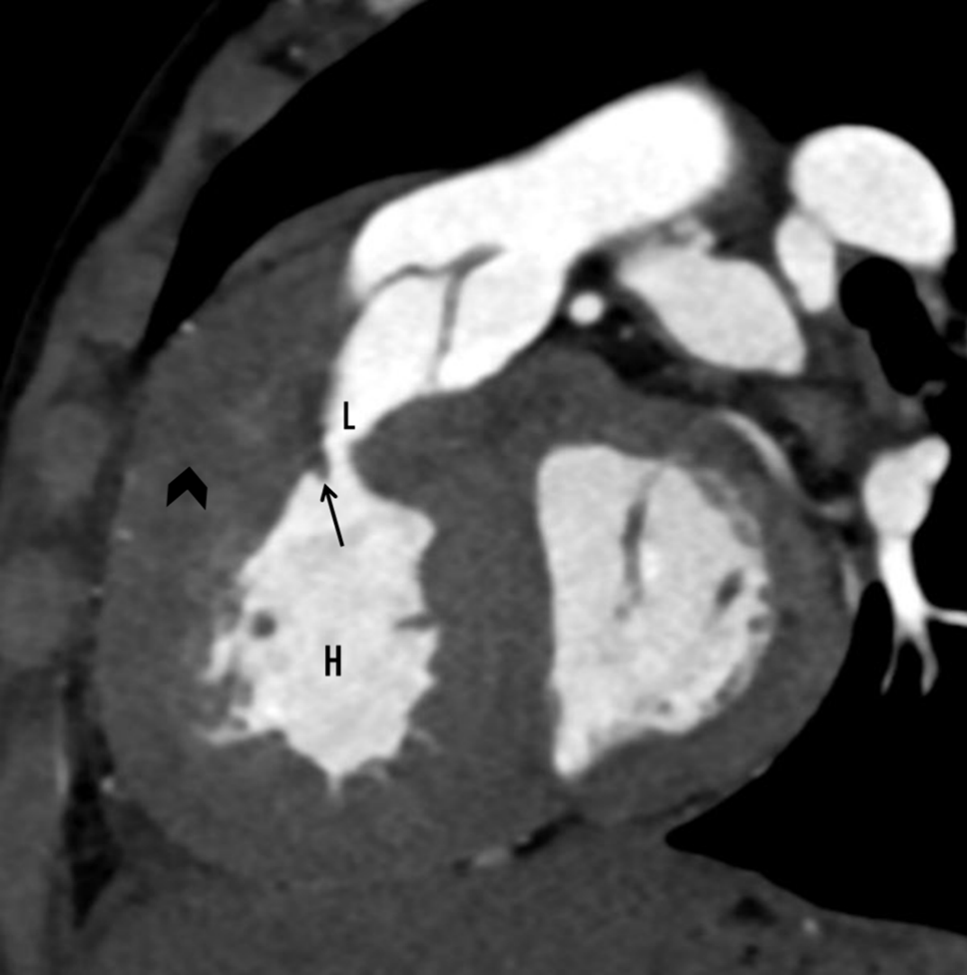
Cardiac CT sagittal section showing a thickened right ventricular wall and infundibulum (chevron) with an anomalous band (thin arrow) running beneath the infundibulum of the outflow tract, which divides the ventricle into high (H) and low (L) pressure chambers

**Fig. 4:**
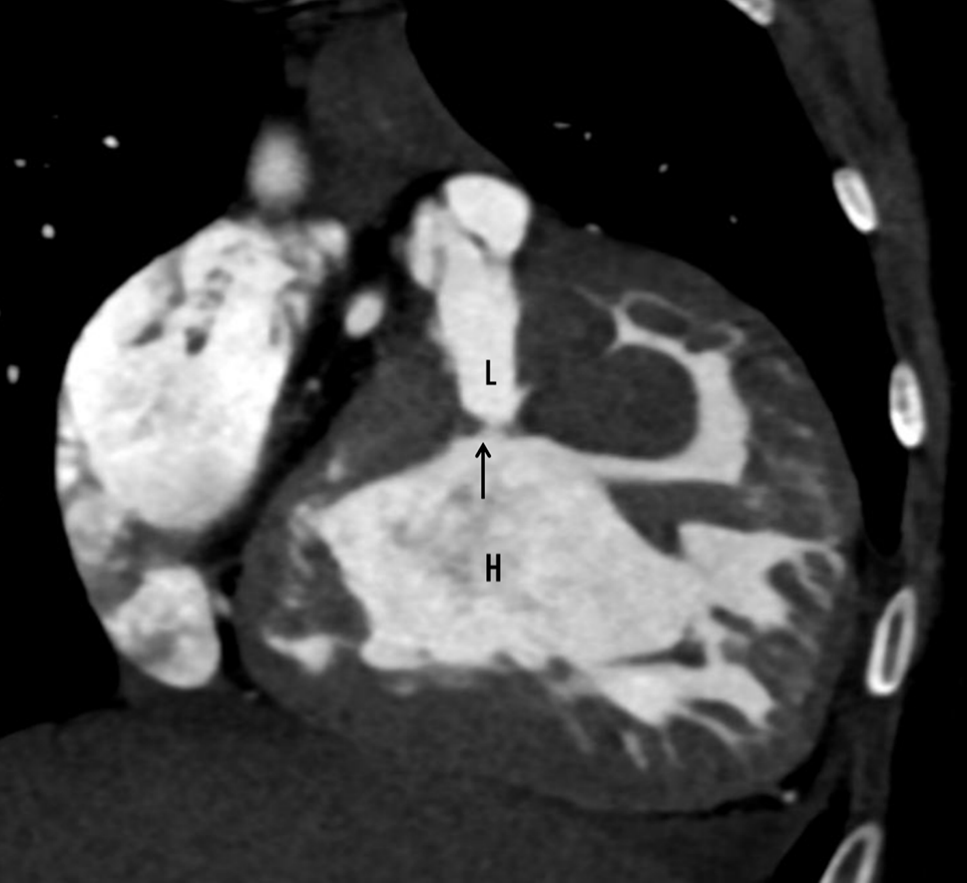
Cardiac CT coronal section showing thickened right ventricular wall with an anomalous band (thin arrow) running beneath the infundibulum of the outflow tract, dividing the ventricle into high (H) and low (L) pressure chambers

**Fig. 5:**
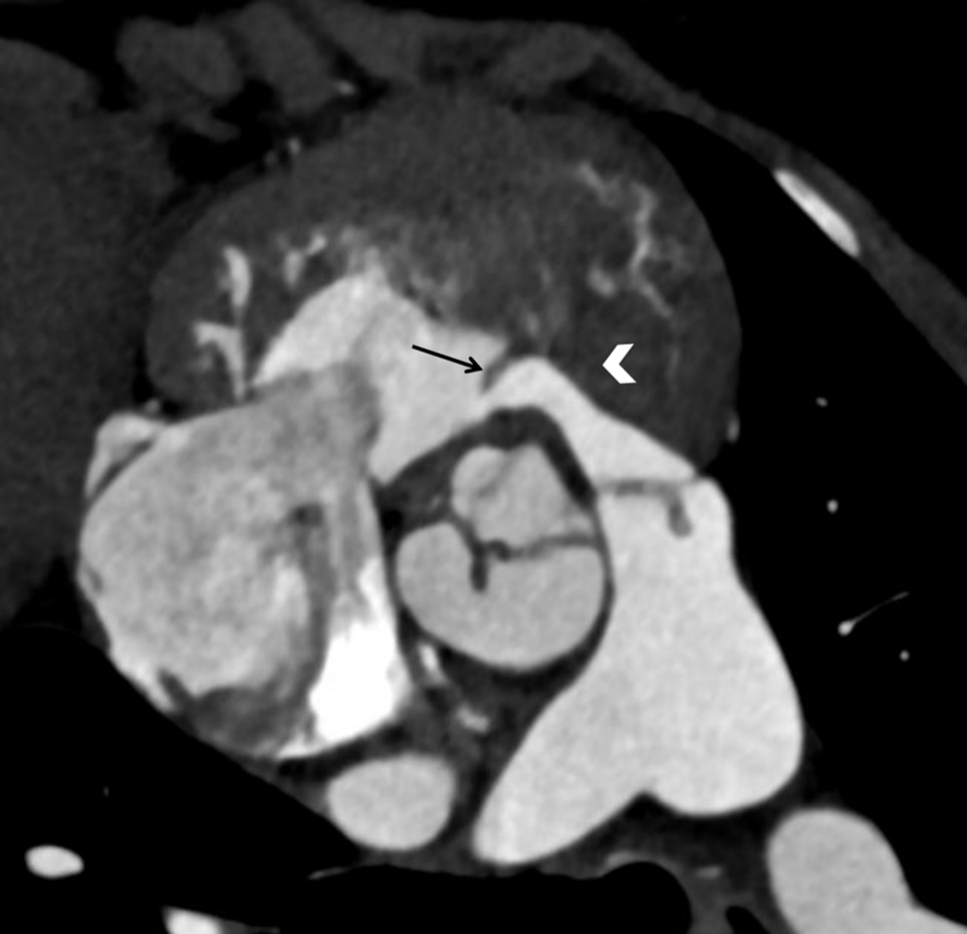
Cardiac CT short axis RVOT reformat showing thickened and hypertrophied infundibulum (chevron) with an anomalous band (thin arrow) running beneath the infundibulum of the outflow tract

**Fig. 6:**
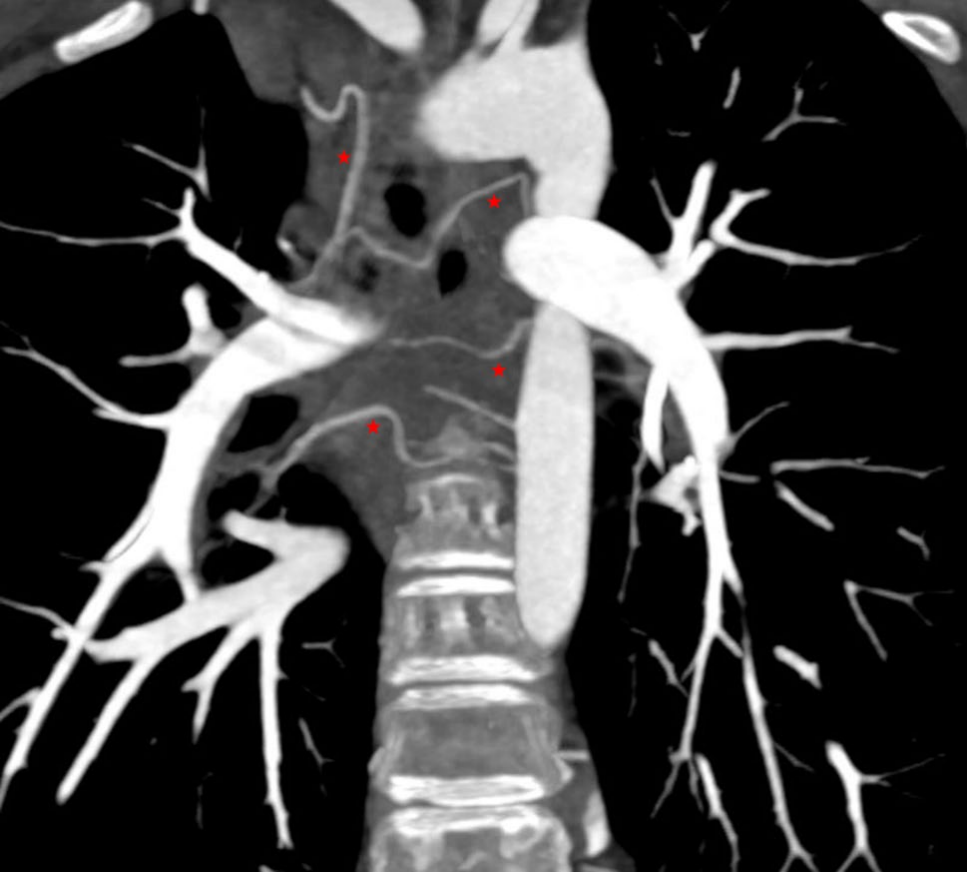
Cardiac CT coronal section, MIP image shows a few major aortopulmonary collaterals (red stars)

**Fig. 7:**
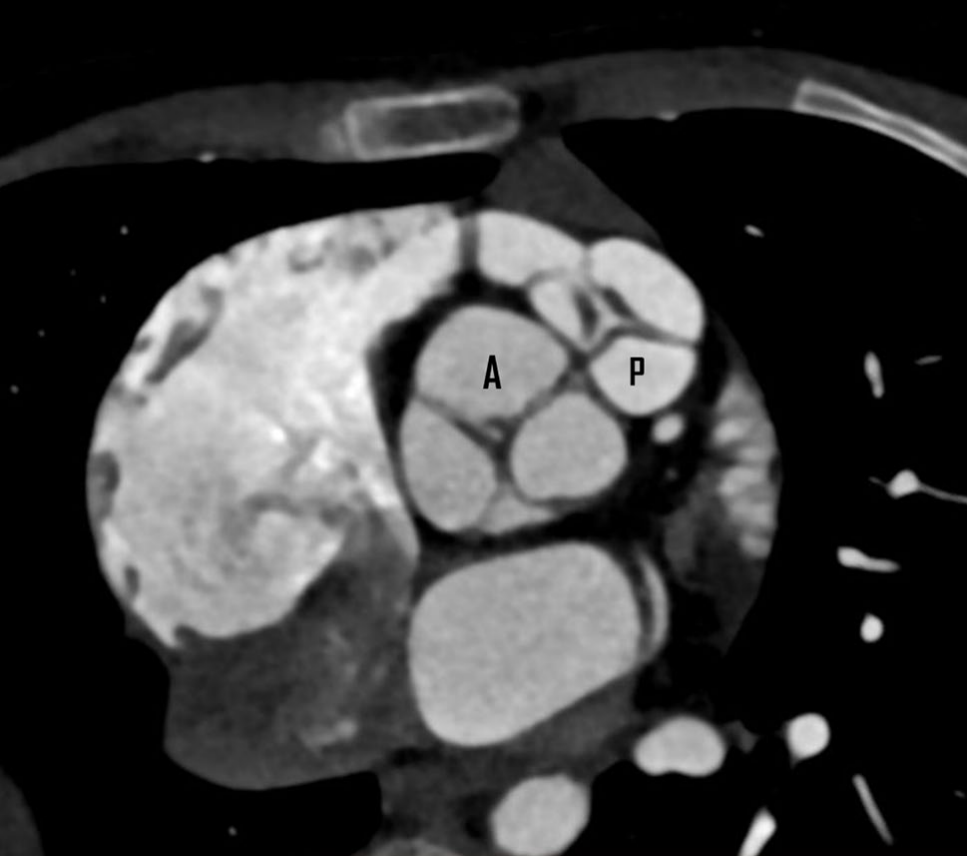
Cardiac CT double oblique image showing thickened aortic valve (A) and pulmonary valve (P) leaflets

CT cardiac angiography was performed on Siemens 256 slice dual-source CT scanner with retrospective ECG gating using 50 ml of 350 mg/dl nonionic iodinated contrast agent. Cardiac CT confirmed the presence of a thin, circumferential subaortic membrane [*, Fig. 1], coursing from the ventricular septum to the anterior mitral leaflet with an eccentric 3 mm fenestration [thin arrow, Fig. 2]. The membrane was seen to cause subvalvular aortic stenosis with resultant left ventricular hypertrophy. CT also revealed dilated right atrium and right ventricle with concentric right ventricular hypertrophy. An anomalous muscular band was seen to arise from the RVOT septal wall and course till the subinfundibular region causing significant luminal stenosis at subinfundibular level [thin arrow in Figs. 3, 4 and 5]. The hypertrophied muscular band divided the right ventricle into a proximal high pressure chamber and a distal low pressure chamber [H, L in Figs. 3 and 4]. Infundibular hypertrophy was seen as well [chevron in Figs. 3 and 5]. Thin major aortopulmonary collateral (MAPCAS) were seen arising from the right internal mammary artery, arch of aorta and the anteromedial aspect of the descending thoracic aorta at D3 level [*, Fig.  6]. Also worth noting was presence of mild aortic and pulmonary valve thickening [A,P in Fig. 7]. Interatrial and interventricular septa were found to be intact. Atrioventricular and ventriculoarterial connections were concordant with thoracic and abdominal situs solitus. Pulmonary and systemic venous drainage was normal.

Based on combined echocardiography and CT findings, a final diagnosis of DCRV with SAM causing both right and left ventricular outflow tract obstruction was made. The patient underwent resection of subaortic membrane with myectomy and RVOT muscle bundle resection along with pericardial patch augmentation.

## Discussion

**Fig. 8:**
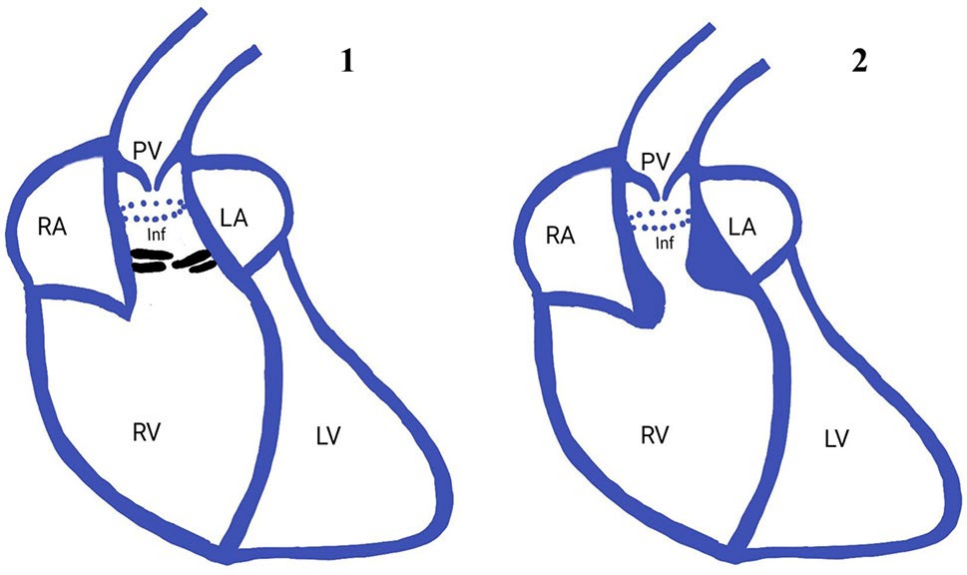
Illustration showing Type 1 and Type 2 double chambered right ventricle

Outflow tract obstruction is most commonly due to valvular causes in adults such as valvular stenosis with subvalvular stenosis encountered less frequently. However in paediatric population, subvalvular stenosis due to congenital anomalies is much more common than in adult population. Double chambered right ventricle, also known as subinfundibular stenosis, is an uncommon congenital heart disease, in which there is a mid-cavitary obstruction dividing the right ventricle into a proximal high pressure chamber and a distal low pressure chamber. It usually presents in childhood but has also been reported in adults as well [7, 8]. Various mechanisms have been proposed behind its etiopathogenesis. One of these says that in the presence of a VSD, the increased blood flow through the defect causes hypertrophy of the supraventricular crest, whereas in the absence of a VSD, superior displacement of the septomarginal trabecula produces progressive hypertrophy of the RVOT. Another proposed mechanism states that DCRV occurs due to extension of a muscular band from the septomarginal trabeculations to the trabecular part of the right ventricle, contributed additionally by some other acquired factors [9]. Based on the type of obstruction, DCRV can be divided into two types [Fig. 8]. Type I DCRV occurs due to presence of an anomalous muscle bundle that crosses the RV chamber while type II DCRV occurs due to endogenous parietal and septal muscle hypertrophy that results in narrowing of the subinfundibular region leading to subinfundibular stenosis. DCRV is commonly associated with VSD which can be observed in up to 90% of cases [10]. However, association with subaortic membrane is extremely rare and only a limited number of cases have been reported in the literature [5, 6, 11]. Interestingly, no VSD was appreciated in our case. Baumstark et al. studied the statistical association between DCRV and SAM and concluded that the actual incidence of combined malformation was 9 times the expected incidence rate on a chance basis [5]. 

**Fig. 9:**
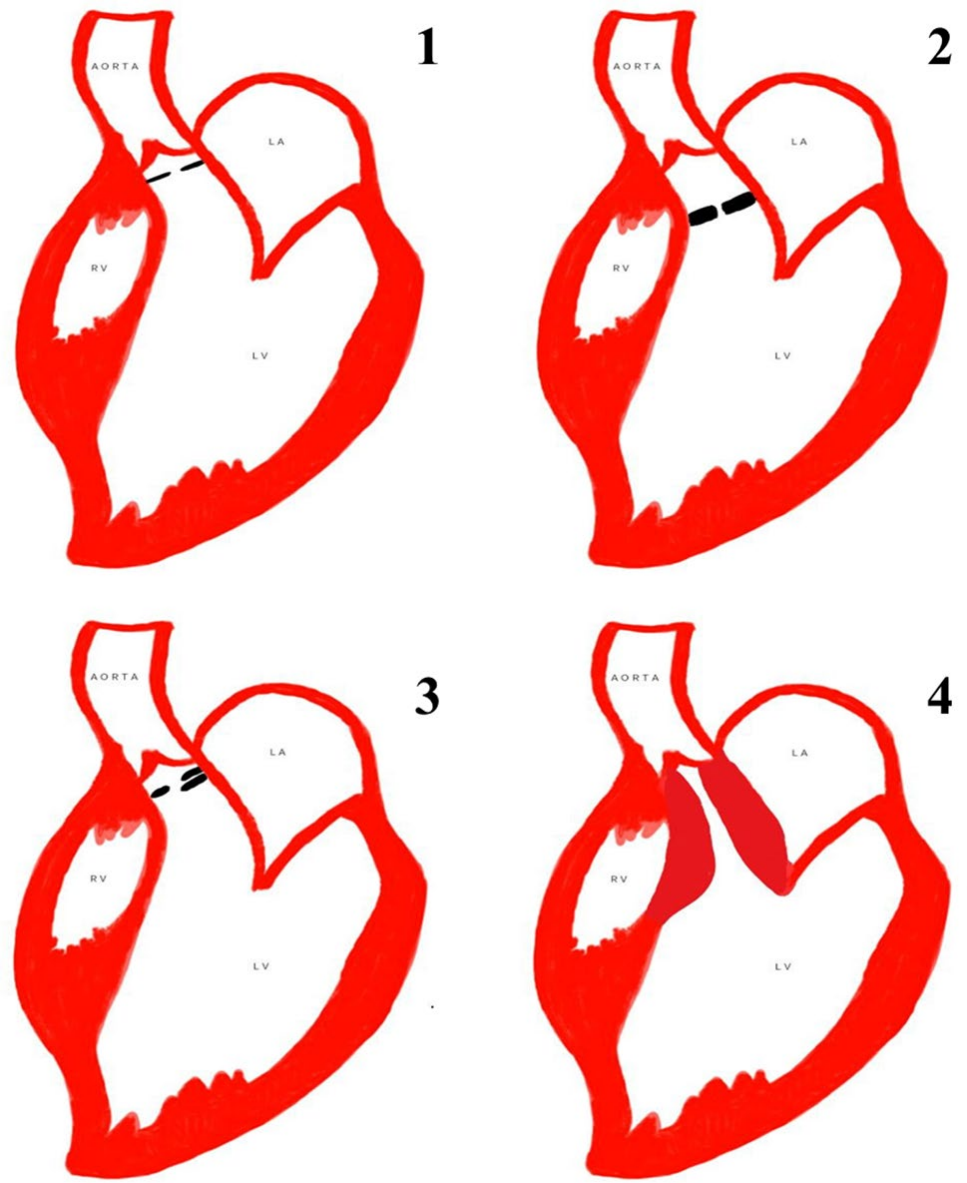
Illustration showing types of subaortic membrane

Subaortic membranes were first described by Keith in 1909 [12]. These are the most common cause of discrete subaortic stenosis and can be classified into various subtypes as illustrated in [Fig. 9]. SAM is a rare pathological entity comprising only 1% of all congenital heart defects but responsible for approximately 15–20% of all obstructive LVOT lesions. These occur predominantly in the first decade of life and their location can vary anywhere from the subvalvular region to further inferior into the left ventricular cavity. The etiopathogenesis behind the development of SAM is a chronic flow disturbance that can result due to anatomical factors such as narrow LVOT, steep atrioventricular septal angle, increased mitral-aortic septation and overriding of aorta. Chronic flow disturbance causes endothelial injury which potentiates smooth muscle proliferation leading to the development of a muscular band at the site. High velocity blood flow also causes injury to the aortic valves. This, along with smooth muscle encroachment from the proliferating muscle bundle, results in thickening and retraction of the aortic valve in the long run. This also explains why aortic regurgitation frequently develops in long standing cases of subaortic membranes and is the most common co-existing condition in SAM [12]. SAM is associated with ventricular septal defects in only 20% cases as opposed to DCRV in which it can be found in up to 90% cases [10]. 

Imaging evaluation with echocardiography and cardiac CT/ MRI is necessary in cases with congenital heart defects not only to identify associated heart diseases but also to predict preoperative complications. Detection of DCRV and SAM is possible using transthoracic echocardiography, however it is not uncommon to miss the diagnosis for various reasons. Failure to visualise subaortic membrane due to a narrow and suboptimal window or misdiagnosing the subvalvular lesion for a valvular pathology are a few of the limitations of transthoracic echocardiography. Sometimes the low pressure chamber in DCRV may be so small that its detection may be missed on transthoracic echocardiography. Transoesophageal echocardiography provides a better window for evaluation and should be preferred in cases where subaortic membrane is suspected. For complete preoperative evaluation, echocardiography is supplemented with cardiac CT which provides both cardiac and extracardiac information and is not operator dependent. On CT, both the anomalous muscle bundle in the right ventricle and the subaortic membrane in the LVOT will show the same attenuation as the myocardium with no abnormal enhancement. Associated finding suggestive of outflow tract obstructive lesions in both chambers includes ventricular hypertrophy. Both DCRV and SAM are progressive in nature producing worsening of symptoms with time. There was also development of MAPCAs in this case. The authors hypothesize progressive subinfundibular stenosis as the cause of MAPCAs in the patient.

## Conclusion

DCRV and SAM are rare cardiac anomalies that can coexist and cause dual chamber obstruction, complicating management in such patients. Cardiac surgery is the definitive treatment and cardiac CT which is a user independent modality helps in the complete evaluation of structural anatomy and defects before planning surgery. A complete preoperative evaluation of congenital heart disease helps identify additional lesions and decide the proper treatment plan which is mandatory for patient care.

### Electronic supplementary material

Below is the link to the electronic supplementary material.

Supplementary Material 1

## Data Availability

Data is provided as supplementary file. The datasets used and/or analysed during the current study available from the corresponding author on reasonablerequest.

## Abbreviations

CT: Computed Tomography
DCRV: Double Chambered Right Ventricle
LVOT: Left Ventricular Outflow Tract
MAPCAS: Major Aorto-Pulmonary Collaterals
RVOT: Right Ventricular Outflow Tract
SAM: Subaortic Membrane
VSD: Ventricular Septal Defect
